# Supplementary material for: Diversity in trap color and height increases species richness of bark and woodboring beetles detected in multiple funnel traps
Source: PLoS One. 2025 May 8;20(5):e0322412. doi: 10.1371/journal.pone.0322412 (PMC12061410; doi:10.1371/journal.pone.0322412)
Supplement: Table S1 — (DOCX) [file pone.0322412.s001.docx]

**Table S1. Source, percentage purity, lure type, and release rate of semiochemical lures used in trapping experiments testing effects of trap color and trap height on detection of bark and wood boring beetles.**

| **Lure** | **Acronym** | **Release device** | **Purity (%)** | **Release rate (mg/day) at 20°C^2^** | **Source** |
| --- | --- | --- | --- | --- | --- |
| Racemic 3-hydroxyhexan-2-one | K6 | Pouch | 99%^1^ | 20–25 | Bedoukian Research Inc., Danbury, CT (chemical); Contech Enterprises Inc., Delta, BC (lures) |
| Racemic 3-hydroxyoctan-2-one | K8 | Pouch | 99%^1^ | 20–25 | Bedoukian Research Inc.(chemical); Contech Enterprises Inc. (lures) |
| Racemic *syn*-2,3-hexanediol | D6 | Pouch | 95%^1^ | 1–2 | Canadian Forest Service, Fredericton, NB (chemical synthesis); Contech Enterprises Inc., (lure) |
| (*E/Z*)-fuscumol | EZF | Rubber septum | 99%^2^ | 0.5–2 | Sylvar Technologies/ Andermatt Canada, Fredericton, NB |
| (*E/Z*)-fuscumol acetate | EZFA | Rubber septum | 99%^2^ | 0.5–2 | Sylvar Technologies/ Andermatt Canada, Fredericton, NB |
| Ethanol | ET | Pouch | 98%^2^ | 300–400 | Contech Enterprises Inc. |

^1^ Determined at Canadian Forest Service, Atlantic Forest Centre, Fredericton, NB

^2^ Supplied by manufacturer
