# Supplementary material for: Diversity in trap color and height increases species richness of bark and woodboring beetles detected in multiple funnel traps
Source: PLoS One. 2025 May 8;20(5):e0322412. doi: 10.1371/journal.pone.0322412 (PMC12061410; doi:10.1371/journal.pone.0322412)
Supplement: Table S2 — Spondylidinae were captured in JI and PO only and Prioninae were captured in GA and JI only. (DOCX) [file pone.0322412.s002.docx]

**Table S2. Results of generalized linear mixed models testing for interactions between site and treatment (treatments = six different combinations of trap height and trap color) on species richness of target taxa captured in multiple funnel traps in Georgia USA (GA), Jilin, China (JI), New Brunswick, Canada (NB), and Białowieża, Poland (PO). Spondylidinae were captured in JI and PO only and Prioninae were captured in GA and JI only.**

| **Response variable** | **Factor** | ***F*** | **df** | ***P*** | **Distribution with best fit** |
| --- | --- | --- | --- | --- | --- |
| **Buprestidae** | Treatment | 14.6 | 5,174 | **<0.001** | Poisson |
|  | Site | 12.7 | 3,174 | **<0.001** |  |
|  | T*S | 2.29 | 15,174 | **0.005** |  |
| **Agrilinae** | Treatment | 95.4 | 5,174 | **<0.001** | Poisson |
|  | Site | 88.1 | 3,174 | **<0.001** |  |
|  | T*S | 1.63 | 14,174 | 0.07 |  |
| **Chrysochroinae** | Treatment | 14.6 | 5,96 | **<0.001** | Gaussian |
|  | Site | 10.4 | 3,96 | **<0.001** |  |
|  | T*S | 0.94 | 15,96 | 0.52 |  |
| **Cerambycidae** | Treatment | 1.41 | 5,174 | 0.22 | Gaussian |
|  | Site | 80.1 | 3,174 | **<0.001** |  |
|  | T*S | 4.54 | 15,174 | **<0.001** |  |
| **Cerambycinae** | Treatment | 1.35 | 5,174 | 0.35 | Gaussian |
|  | Site | 176 | 3,174 | **<0.001** |  |
|  | T*S | 3.94 | 15,174 | **<0.001** |  |
| **Lamiinae** | Treatment | 3.37 | 5,174 | **0.006** | Gaussian |
|  | Site | 34.2 | 3,174 | **<0.001** |  |
|  | T*S | 2.34 | 15,174 | **0.004** |  |
| **Lepturinae** | Treatment | 3.05 | 5,174 | **0.01** | Poisson |
|  | Site | 24.1 | 3,174 | **<0.001** |  |
|  | T*S | 3.02 | 15,174 | **<0.001** |  |
| **Spondylidinae** | Treatment | 10.3 | 5,72 | **<0.001** | Gaussian |
|  | Site | 27.6 | 1,72 | **<0.001** |  |
|  | T*S | 5.09 | 5,72 | **<0.001** |  |
| **Prioninae** | Treatment | 3.38 | 5,90 | **0.008** | Gaussian |
|  | Site | 4.53 | 1,90 | **0.04** |  |
|  | T*S | 4.80 | 5,90 | **<0.001** |  |
| **Scolytinae** | Treatment | 4.01 | 5,174 | **0.002** | Poisson |
|  | Site | 90.9 | 3,174 | **<0.001** |  |
|  | T*S | 0.56 | 15,174 | 0.89 |  |
| **All target taxa** | Treatment | 4.00 | 5,174 | **<0.002** | Gaussian |
|  | Site | 66.3 | 3,174 | **<0.001** |  |
|  | T*S | 3.89 | 15,174 | **<0.001** |  |
