# Supplementary material for: Diversity in trap color and height increases species richness of bark and woodboring beetles detected in multiple funnel traps
Source: PLoS One. 2025 May 8;20(5):e0322412. doi: 10.1371/journal.pone.0322412 (PMC12061410; doi:10.1371/journal.pone.0322412)
Supplement: Table S4 — (DOCX) [file pone.0322412.s004.docx]

**Table S4. Results of generalized linear models testing for the effects of trap height, trap color and their interaction on species richness of target taxa by family and subfamily captured in traps at four sites: Georgia, USA (GA), Jilin, China (JI), New Brunswick, Canada (NB), and Białowieża, Poland (PO). Data were analyzed separately by site when whenever the preliminary analyses found a significant interaction between site and trap color-height treatment (see Table S2).**

| **Taxa** | **Site(s)** | **Factor** | ***F*** | **d.f.** | ***P*** | **Distribution with best fit** |
| --- | --- | --- | --- | --- | --- | --- |
| **Buprestidae** | GA | Height | 5.00 | 1,48 | **0.030** | Poisson |
|  |  | Color | 2.27 | 2,48 | 0.114 |  |
|  |  | H*C | 3.90 | 2,48 | **0.027** |  |
|  | JI | Height | 0.38 | 1,42 | 0.540 | Poisson |
|  |  | Color | 2.89 | 2,42 | 0.067 |  |
|  |  | H*C | 2.55 | 2,42 | 0.091 |  |
|  | NB | Height | 3.35 | 1,42 | 0.075 | Poisson |
|  |  | Color | 5.41 | 2,42 | **0.021** |  |
|  |  | H*C | 3.99 | 2,42 | 0.497 |  |
|  | PO | Height | 8.69 | 1,42 | **0.005** | Poisson |
|  |  | Color | 10.9 | 2,42 | **<0.001** |  |
|  |  | H*C | 0.70 | 2,42 | 0.503 |  |
| Agrilinae | GA, JI, | Height | 3.28 | 1,189 | 0.072 | Poisson |
|  | NB, PO | Color | 34.6 | 2,189 | **<0.001** |  |
|  |  | H*C | 4.63 | 2,189 | **0.011** |  |
|  |  | Site | 4.09 | 3,189 | **0.008** |  |
| Chrysochroinae | GA, JI, | Height | 8.56 | 1,111 | **0.004** | Gaussian |
|  | NB, PO | Color | 36.6 | 2,111 | **<0.001** |  |
|  |  | H*C | 6.43 | 2,111 | **0.002** |  |
|  |  | Site | 9.34 | 3,111 | **<0.001** |  |
| **Cerambycidae** | GA | Height | 4.61 | 1,48 | **0.037** | Gaussian |
|  |  | Color | 1.12 | 2,48 | 0.334 |  |
|  |  | H*C | 3.24 | 2,48 | **0.048** |  |
|  | JI | Height | 1.67 | 1,42 | 0.203 | Gaussian |
|  |  | Color | 11.3 | 2,42 | **<0.001** |  |
|  |  | H*C | 1.93 | 2,42 | 0.158 |  |
|  | NB | Height | 0.12 | 1,42 | 0.727 | Poisson |
|  |  | Color | 3.28 | 2,42 | **0.047** |  |
|  |  | H*C | 0.18 | 2,42 | 0.838 |  |
|  | PO | Height | 8.38 | 1,42 | **0.006** | Gaussian |
|  |  | Color | 4.49 | 2,42 | **0.017** |  |
|  |  | H*C | 3.73 | 2,42 | **0.032** |  |
| Cerambycinae | GA | Height | 0.15 | 1,48 | 0.701 | Gaussian |
|  |  | Color | 2.55 | 2,48 | 0.089 |  |
|  |  | H*C | 1.37 | 2,48 | 0.265 |  |
|  | JI | Height | 0.56 | 1,42 | 0.457 | Gaussian |
|  |  | Color | 8.20 | 2,42 | **0.001** |  |
|  |  | H*C | 1.17 | 2,42 | 0.320 |  |
|  | NB | Height | 0.25 | 1,42 | 0.621 | Poisson |
|  |  | Color | 0.49 | 2,42 | 0.618 |  |
|  |  | H*C | 0.50 | 2,42 | 0.953 |  |
|  | PO | Height | 32.2 | 1,42 | **<0.001** | Gaussian |
|  |  | Color | 0.69 | 2,42 | 0.509 |  |
|  |  | H*C | 0.99 | 2,42 | 0.378 |  |
| Lamiinae | GA | Height | 2.89 | 1,48 | 0.095 | Gaussian |
|  |  | Color | 1.11 | 2,48 | 0.339 |  |
|  |  | H*C | 2.04 | 2,48 | 0.142 |  |
|  | JI | Height | 4.58 | 1,42 | **0.038** | Gaussian |
|  |  | Color | 1.00 | 2,42 | 0.377 |  |
|  |  | H*C | 0.79 | 2,42 | 0.459 |  |
|  | NB | Height | 3.73 | 1,42 | 0.060 | Gaussian |
|  |  | Color | 1.81 | 2,42 | 0.177 |  |
|  |  | H*C | 1.99 | 2,42 | 0.149 |  |
|  | PO | Height | 8.97 | 1,42 | 0.005 | Gaussian |
|  |  | Color | 6.43 | 2,42 | **0.004** |  |
|  |  | H*C | 3.17 | 2,42 | **0.052** |  |
| Lepturinae | GA | Height | 7.20 | 1,48 | **0.010** | Poisson |
|  |  | Color | 1.35 | 2,48 | 0.268 |  |
|  |  | H*C | 0.23 | 2,48 | 0.793 |  |
|  | JI | Height | 0.05 | 1,42 | 0.825 | Poisson |
|  |  | Color | 6.32 | 2,42 | **0.004** |  |
|  |  | H*C | 0.62 | 2,42 | 0.542 |  |
|  | NB | Height | 0.19 | 1,42 | 0.667 | Poisson |
|  |  | Color | 5.11 | 2,42 | **0.010** |  |
|  |  | H*C | 1.97 | 2,42 | 0.152 |  |
|  | PO | Height | 0.06 | 1,42 | 0.802 | Poisson |
|  |  | Color | 6.33 | 2,42 | **0.004** |  |
|  |  | H*C | 0.96 | 2,42 | 0.390 |  |
| Spondylidinae | JI | Height | 0.30 | 1,30 | 0.348 | Poisson |
|  |  | Color | 0.30 | 2,30 | 0.742 |  |
|  |  | H*C | 0.30 | 2,30 | 0.742 |  |
|  | PO | Height | 73.5 | 1,42 | **<0.001** | Gaussian |
|  |  | Color | 2.98 | 2,42 | 0.062 |  |
|  |  | H*C | 0.54 | 2,42 | 0.589 |  |
| Prioninae | GA | Height | 0.99 | 1,48 | 0.326 | Gaussian |
|  |  | Color | 6.35 | 2,48 | **0.004** |  |
|  |  | H*C | 1.54 | 2,48 | 0.225 |  |
|  | JI | Height | 26.5 | 1,42 | **<0.001** | Gaussian |
|  |  | Color | 0.41 | 2,42 | 0.669 |  |
|  |  | H*C | 0.95 | 2,42 | 0.396 |  |
| **Scolytinae** | GA, JI, NB, | Height | 20.7 | 1,189 | **<0.001** | Poisson |
|  | PO | Color | 1.05 | 2,189 | 0.353 |  |
|  |  | H*C | 0.44 | 2,189 | 0.643 |  |
|  |  | Site | 92.8 | 3,189 | **<0.001** |  |
| **All target taxa** | GA | Height | 9.08 | 1,48 | **0.004** | Gaussian |
|  |  | Color | 5.37 | 2,48 | 0.290 |  |
|  |  | H*C | 3.87 | 2,48 | 0.304 |  |
|  | JI | Height | 4.32 | 1,42 | **0.044** | Gaussian |
|  |  | Color | 6.43 | 2,42 | **0.004** |  |
|  |  | H*C | 3.66 | 2,42 | **0.034** |  |
|  | NB | Height | 1.33 | 1,42 | 0.256 | Gaussian |
|  |  | Color | 2.99 | 2,42 | 0.061 |  |
|  |  | H*C | 0.12 | 2,42 | 0.885 |  |
|  | PO | Height | 3.79 | 1,42 | 0.058 | Poisson |
|  |  | Color | 6.20 | 2,42 | **0.004** |  |
|  |  | H*C | 3.01 | 2,42 | 0.060 |  |
|  |  |  |  |  |  |  |
