# Supplementary material for: Diversity in trap color and height increases species richness of bark and woodboring beetles detected in multiple funnel traps
Source: PLoS One. 2025 May 8;20(5):e0322412. doi: 10.1371/journal.pone.0322412 (PMC12061410; doi:10.1371/journal.pone.0322412)
Supplement: Table S6 — (DOCX) [file pone.0322412.s006.docx]

**Table S6. Sample completeness and estimated species richness of bark and wood boring beetles (Buprestidae, Cerambycidae, Scolytinae) detected in the entire sample of 48–54 pheromone-baited multiple funnel traps deployed in Georgia, USA, Jilin, China, New Brunswick, Canada, and Białowieża, Poland.**

| **Site** | **No. traps** | **SC** | **Species Richness** | | | **CBE Cmax=0.988** |
| --- | --- | --- | --- | --- | --- | --- |
|  |  |  | **Ob** | **AE (95% CI)** | **CBE (Cmax)** |  |
| Georgia | 54 | 0.84 | 136 | 162 (139–185) | 153 (0.994) | 143 |
| Jilin | 48 | 0.81 | 82 | 102 (82–122) | 95 (0.990) | 94 |
| New Brunswick | 48 | 0.74 | 60 | 81 (53–108) | 70 (0.988) | 70 |
| Poland | 48 | 0.82 | 87 | 107 (84–129) | 99 (0.992) | 96 |

Equal numbers of black, green, and purple traps were deployed in the understory and canopy at each site. SC = Sample completeness at q = 0, i.e., the maximum proportion of total species that was detected by the sample, e.g., the minimum estimated percentage of species present at a site that were not detected by the sample ranged from 16% in Georgia to 36% in New Brunswick. Ob = Observed number of species detected; AE = Asymptotic-based estimate of true species richness with 95% confidence interval, based on sample size; CBE = Coverage-based estimate of true species richness standardized for a maximum coverage of 0.988 to allow fair comparison of relative species richness between the sites. All parameters were estimated using the iNEXT 4-steps program.
