# Supplementary figures and images for: Diversity in trap color and height increases species richness of bark and woodboring beetles detected in multiple funnel traps
Source: PLoS One. 2025 May 8;20(5):e0322412. doi: 10.1371/journal.pone.0322412 (PMC12061410; doi:10.1371/journal.pone.0322412)

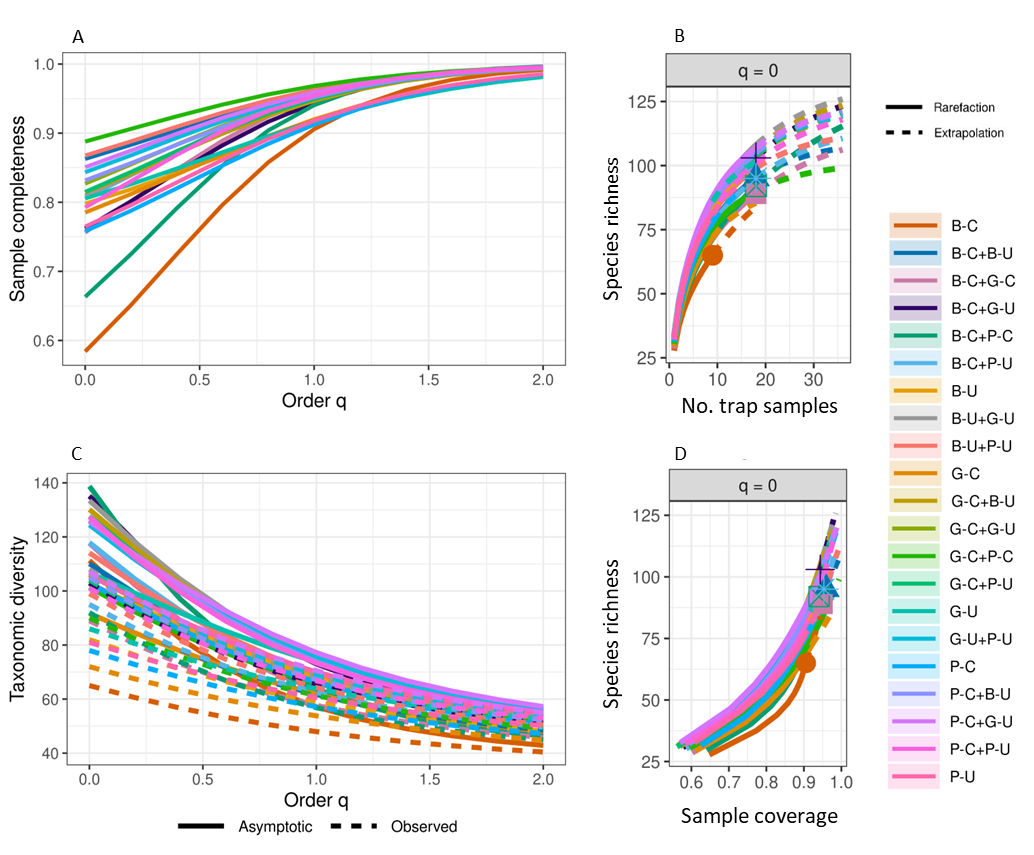

Supplement: Fig S1 — Legend: Each treatment was replicated 9 times for a total of 54 traps. A) sample completeness: values at q = 0 estimate the proportion of total species present at a site that were detected in the sample; B) species richness vs. number of trap samples, estimated by rarefaction or extrapolation based on sample size; C) observed and asymptotic species richness estimates of species diversity (when q = 0); and D) coverage-based species richness for standardized coverage value of Cmax = 0.944. B = black, G = green, P = purple, C = canopy, U = understory. (TIF) [file pone.0322412.s007.tif]

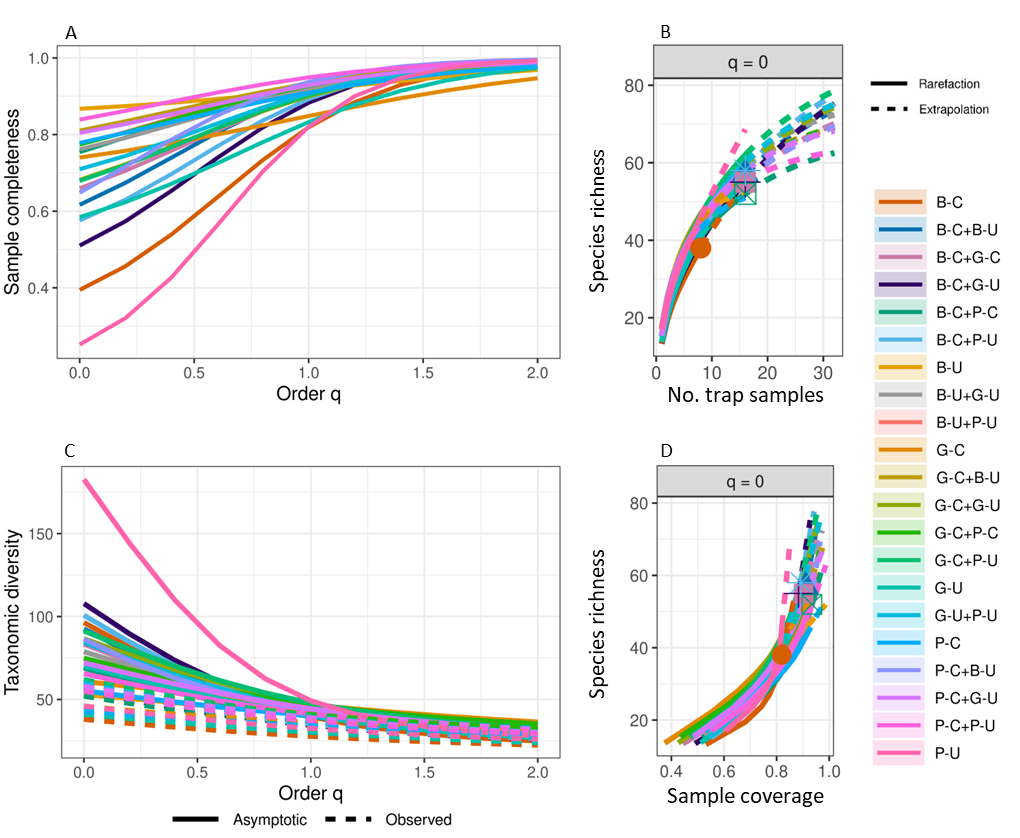

Supplement: Fig S2 — Legend: Each treatment was replicated 8 times for a total of 48 traps. A) sample completeness: values at q = 0 estimate the proportion of total species present at a site that were detected in the sample; B) species richness vs. number of trap samples, estimated by rarefaction or extrapolation based on sample size; C) observed and asymptotic species richness estimates of species diversity (when q = 0); and D) coverage-based species richness for standardized coverage value of Cmax = 0.923. B = black, G = green, P = purple, C = canopy, U = understory. (TIF) [file pone.0322412.s008.tif]

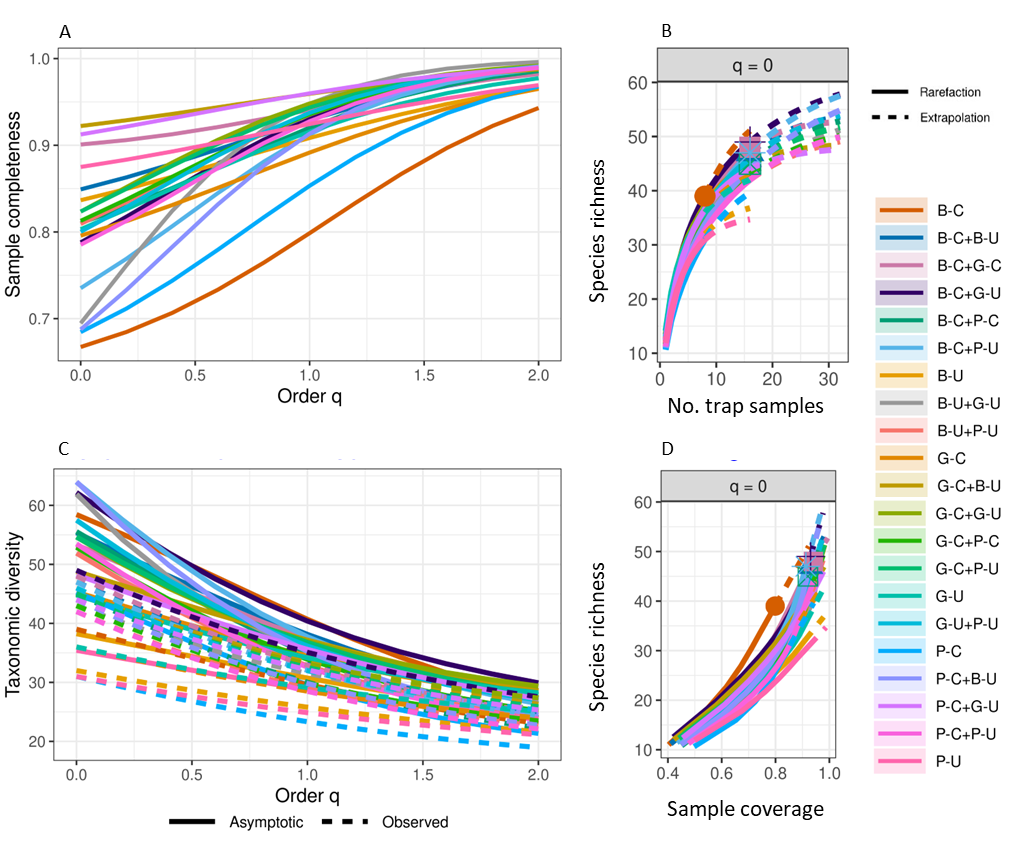

Supplement: Fig S3 — Legend: Each treatment was replicated 8 times for a total of 48 traps. A) sample completeness: values at q = 0 estimate the proportion of total species present at a site that were detected in the sample; B) species richness vs. number of trap samples, estimated by rarefaction or extrapolation based on sample size; C) observed and asymptotic species richness estimates of species diversity (when q = 0); and D) coverage-based species richness for standardized coverage value of Cmax = 0.85. B = black, G = green, P = purple, C = canopy, U = understory. (TIF) [file pone.0322412.s009.tif]

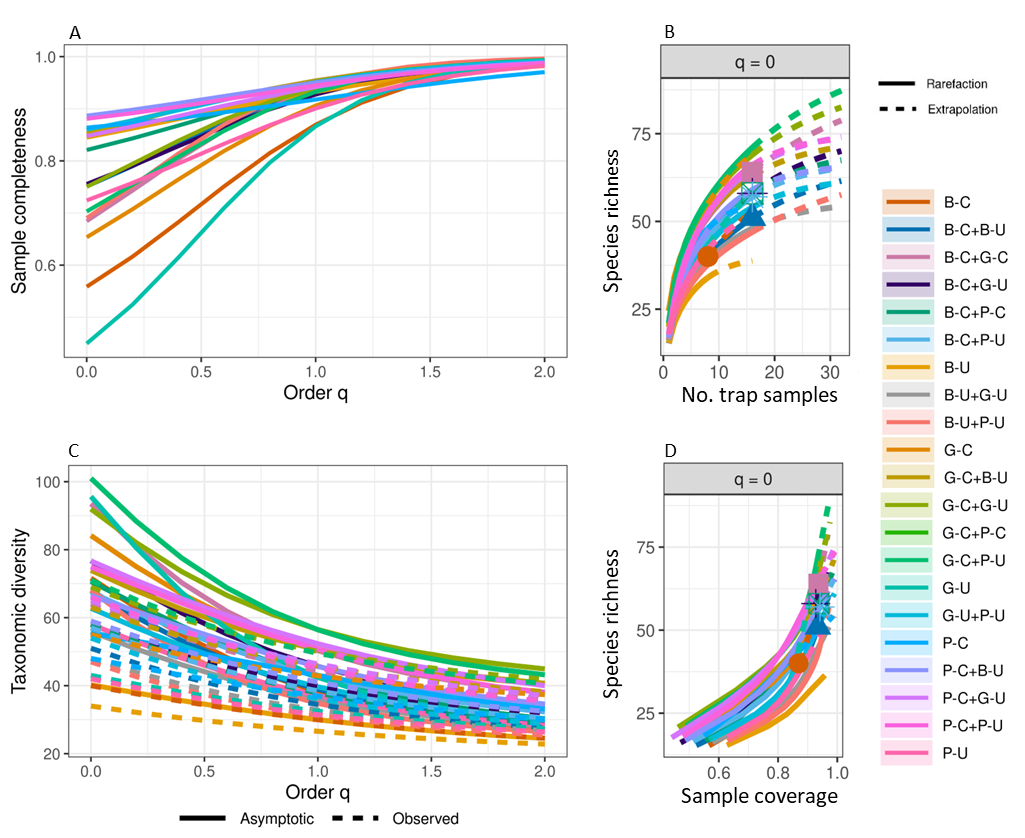

Supplement: Fig S4 — Legend: Each treatment was replicated 8 times for a total of 48 traps. A) sample completeness: values at q = 0 estimate the proportion of total species present at a site that were detected in the sample; B) species richness vs. number of trap samples, estimated by rarefaction or extrapolation based on sample size; C) observed and asymptotic species richness estimates of species diversity (when q = 0); and D) coverage-based species richness for standardized coverage value of Cmax = 0.906. B = black, G = green, P = purple, C = canopy, U = understory. (TIF) [file pone.0322412.s010.tif]

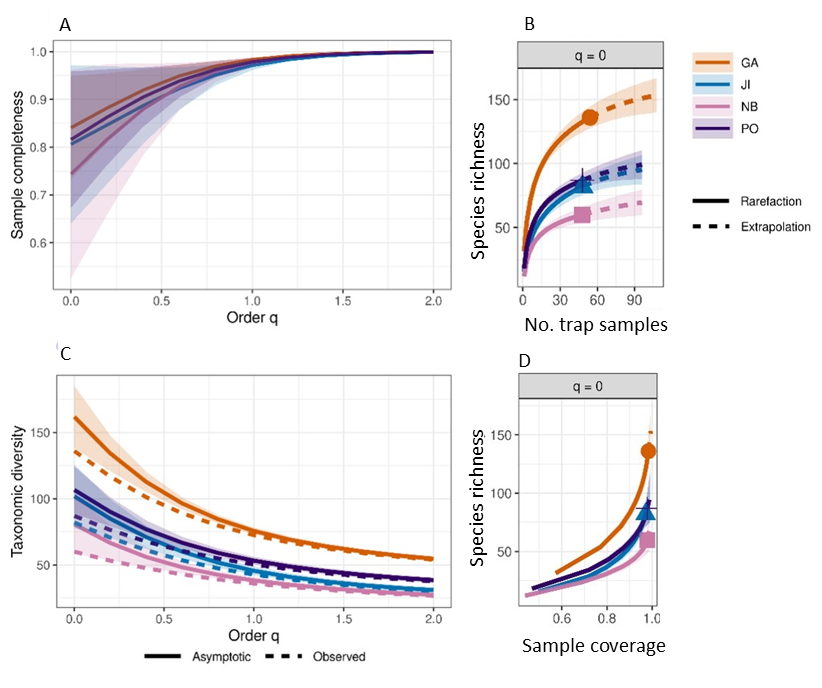

Supplement: Fig S5 — Legend: Equal numbers of black, green, and purple traps were deployed in the understory and canopy at each site. A) sample completeness: values at q = 0 estimate the proportion of total species present at a site that were detected in the sample; B) species richness vs. number of trap samples, estimated by rarefaction or extrapolation based on sample size; C) observed and asymptotic species richness estimates of species diversity (when q = 0); and D) coverage-based species richness for standardized coverage value of Cmax = 0.99. (TIF) [file pone.0322412.s011.tif]
