# Supplementary material for: Diversity in trap color and height increases species richness of bark and woodboring beetles detected in multiple funnel traps
Source: PLoS One. 2025 May 8;20(5):e0322412. doi: 10.1371/journal.pone.0322412 (PMC12061410; doi:10.1371/journal.pone.0322412)
Supplement: Attachment — Submitted filename: review_Galko.pdf [file pone.0322412.s012.pdf]

Peer Review of Manuscript: *"Diversity in Trap Color and Height Increases Species Richness of Bark and Woodboring Beetles Detected in Multiple Funnel Traps"*

Overall Assessment:

The manuscript presents a well-structured and methodologically sound study on optimizing the detection of bark and woodboring beetles (BBWB) through the use of different trap colors and heights. The research is highly relevant for forest protection and invasive species management, providing insights with practical applications. The study's multinational scope (Canada, Poland, USA, China) and standardized methodology enhance its significance. The findings highlight how varied trap configurations improve species detection, offering valuable guidance for biosecurity and ecological monitoring programs.

Strengths:

1. **Scientific and Practical Significance:** The study addresses a critical aspect of invasive species monitoring, demonstrating how trap color and height influence detection efficiency.
2. **Robust Experimental Design:** The use of multiple funnel traps with controlled variables (color, height, lure treatment) ensures reliable data collection.
3. **Comprehensive Statistical Analysis:** Techniques such as GLMM, perMANOVA, and species accumulation curves strengthen the credibility of the results.
4. **Broad Geographical Scope:** Data from diverse regions allow for cross-site comparisons, increasing the study's applicability.
5. **Potential for Practical Implementation:** The findings contribute to optimizing forest pest monitoring strategies.

Suggested Improvements (Minor Revisions):

1. **Introduction**
   - The introduction could benefit from a clearer statement of the research question. The authors should also provide a more concise overview of the literature on trap color and height preferences of BBWB.
   - Additionally, given the overall length of the manuscript, the introduction is relatively short, and the topic should be expanded further.
   - Sentences like *"We used…."* or *"We replicated…"* (lines 106–113) do not fit well within the Introduction section.
2. **Ecological Justification for Trap Colors:**
   - While the selection of black, green, and purple traps is methodologically sound, a clearer discussion of how these colors relate to beetle visual ecology (e.g., photoreceptor spectral sensitivity, background contrast) would enhance the study's ecological context.
3. **Site-Specific Environmental Considerations:**
   - Differences in trap efficiency across sites may be influenced by local conditions (e.g., canopy cover, light availability, host tree composition). Including a discussion on environmental factors affecting beetle attraction would strengthen the interpretation.
   - The trap placement heights varied significantly across different countries.
4. **Clarification of Methodological Details:**
   - Specify the exact placement of "canopy" traps in relation to tree height and structure.

- Indicate whether sampling intervals aligned with peak beetle activity periods.
- The UHR ethanol lure should have been installed earlier, as many species of ambrosia beetles are active as early as March–April.

5. **Statistical and Data Reporting:**
   - Ensure consistency in citation of statistical methods and software (e.g., R packages used for analyses are listed in References (89) but not mentioned in the Methodology section).
   - Clarify whether multiple testing corrections (e.g., FDR adjustments) were applied to pairwise comparisons.
6. **Reference and Citation Consistency:**
   - Verify that all in-text citations have corresponding entries in the reference list.
   - Standardize taxonomic names and ensure proper formatting (italicization of genus/species epithets).

Conclusion:

This manuscript provides valuable insights into the effectiveness of multi-colored and multi-height trapping strategies for detecting BBWB. The study is well-conceived and methodologically robust, with findings that have direct applications in invasive species management. The suggested minor revisions will further enhance the clarity and ecological context of the manuscript. I recommend its publication after minor revisions.
